# Investigation of Indigenous Arbuscular Mycorrhizal Performance Using a *Lotus japonicus* Mycorrhizal Mutant

**DOI:** 10.3390/plants9050658

**Published:** 2020-05-22

**Authors:** Taisuke Teranishi, Yoshihro Kobae

**Affiliations:** Laboratory of Crop Nutrition, Department of Sustainable Agriculture, Rakuno Gakuen University, Ebetsu, Hokkaido 069-8501, Japan; tt04026900@gmail.com

**Keywords:** arbuscular mycorrhizal fungi (AMF), exotic AMF inoculant, indigenous AMF, *Lotus japonicus* mycorrhizal mutant

## Abstract

Most plants are usually colonized with arbuscular mycorrhiza fungi (AMF) in the fields. AMF absorb mineral nutrients, especially phosphate, from the soil and transfer them to the host plants. Inoculation with exotic AMF is thought to be effective when indigenous AMF performance is low; however, there is no method for evaluating the performance of indigenous AMF. In this study, we developed a method to investigate the performance of indigenous AMF in promoting plant growth. As *Lotus japonicus* mutant (*str*) that are unable to form functional mycorrhizal roots were considered to be symbiosis negative for indigenous mycorrhizal performance, we examined the growth ratios of wild-type and *str* mycorrhizal mutant using 24 soils. Each soil had its own unique indigenous mycorrhizal performance, which was not directly related to the colonization level of indigenous AMF or soil phosphate level. The low indigenous mycorrhizal performance could not be compensated by the inoculation of exotic AMF. Importantly, indigenous mycorrhizal performance was never negative; however, the inoculation of exotic AMF into the same soil led to both positive and negative performances. These results suggest that indigenous mycorrhizal performance is affected by soil management history and is basically harmless to the plant.

## 1. Introduction

Most plants, including many economically important crops, are usually colonized with arbuscular mycorrhiza fungi (AMF) in the subphylum Glomeromycotina [1] in the fields. AMF colonize roots to obtain carbon sources and develop extraradical hyphae that absorb mineral nutrients from the soil and transfer them to the host plants [2]. AMF that have achieved a symbiosis that is sufficient to amplify their own biomass often form spores in the soils and develop intraradical mycelia with many vesicles, although these hyphal morphologies depend on the AMF type [3,4]. These vegetative and reproductive AMF hyphal structures serve as a source of inoculation for colonizing different plant species in fields, because AMF generally lack strict host specificity; accordingly, the root is often co-colonized with multiple AMF species [5,6].

To investigate the biological properties of each AMF species, many cultured lines have been established by isolating spores and inoculating a single spore in plants in a pot culture or axenic root organ culture [7]; however, they have been generated using only limited species [8]. Inoculation studies of these lines in different plant species, with the exception of modern wheat or barley varieties [9,10], have led to one well-recognized conclusion, i.e., AMF colonization is, in many cases, beneficial for plant nutrition and productivity (see the objective review [11]). Moreover, pot inoculation studies have shown that phosphate (P) uptake by the host plant is often improved and the P concentration in shoots is also increased in the mycorrhized condition compared with the non-mycorrhized condition [3]. This improvement in P uptake led to an expectation that AMF inoculants can be used as bio-fertilizers; i.e., that AMF inoculation in the field may enable farmers to decrease the amount of P fertilizers [12]. For example, inoculation of AMF can substantially reduce P fertilizer application to Welsh onions and lead to the achievement of a marketable yield under field conditions [13]; however, it remains debatable whether mycorrhizal fungi increase the transport of P to plants directly [14]. One way to utilize AM symbiosis in crop cultivation is to increase the propagules of indigenous AMF in the soil. The increased AMF propagules in the soil after the cultivation of host crops can improve the productivity of soybean crops in the following year compared with the former cultivation of non-host crops [15,16,17]. Accordingly, it is expected that P fertilization of the next-year cultivation can be reduced by half [18]. Another approach consists in inoculating the soil with exotic AMF culture lines. As mentioned above, it has been proven that AMF inoculation is useful in many cases, at least in pot tests under well-controlled environmental conditions. However, it is also true that not all inoculation tests provide positive results, even in pot experiments; some plant species clearly show a positive effect of the inoculation of AMF, while others show neutral or even negative effects of the same AMF [19]. The outcome of these mycorrhization approaches is considered to be context dependent [20]; i.e., the type of AMF, plant type, growth condition, growth stage, soil abiotic (nutritional and physical) and biotic (indigenous AMF and soil microbes) properties, etc., affect the performance of mycorrhizas [21,22,23].

Whether the AMF inoculation strategy is effective or not remains a matter of debate [8,11,24]. Usually, the soil contains a large amount of AMF propagules [25,26]. In addition, it has been suggested that indigenous AMF are better adapted to the local edaphic conditions than are exotic AMF and are, thus, better able to promote plant growth [27,28]. In fact, there are few examples of increased crop yields after AMF inoculation in the field [29]. However, it is possible that the performance of indigenous AMF is severely decreased in some soils because of various changes in the soil management history (e.g., excessive tillage, sterilization, and fertilization) [30,31]. Therefore, if the low performance of indigenous AMF could be investigated in advance, the inoculation of exotic AMF could also be effective [32].

To the best of our knowledge, there is no method for evaluating the performance of indigenous AMF. In this study, we developed a method to evaluate the performance of indigenous AMF in promoting plant growth in pot culture. We used a mycorrhizal mutant of *Lotus japonicus*, MG-20, which is a model plant of legumes. The mutant line has a nonsense mutation in the *STR* (*stunted arbuscule*) gene, which encodes an ABC transporter [33]. *STR* is thought to be implicated in lipid transfer from plants to AMF, as assessed using genetic analyses [34,35,36,37]. Recent genome analyses have revealed that AMFs are dependent on plants for lipid synthesis, which is essential for the establishment of symbiosis [38,39]. Thus, in the *str* mutant of *L. japonicus*, *Medicago trucatula*, and rice (*Oryza sativa*), the early root-colonization stage looks normal, but the branching of arbuscules, which are intracellular symbiotic fungal structures that play a central role in mycorrhizal function, is severely inhibited (*stunted arbuscule*: *str*) [33,40,41]. The expression of mycorrhiza-specific P and ammonium transporter genes in the *L. japonicus str* mutant is severely inhibited compared with wild-type (WT) plants [33], suggesting the attenuation of a broad range of mycorrhizal functions in this mutant. From a different perspective, if the growth of WT plants under the presence of AMF is similar to that of *str*, the effect of colonization with indigenous AMF under the assay condition is not expressed (i.e., low performance). Thus, we examined the growth ratios of WT and *str* mycorrhizal mutant using 24 soils. Furthermore, we investigated whether an AMF inoculum was effective in promoting plant growth in soils that were assessed as having low indigenous mycorrhizal performance.

## 2. Materials and Methods

### 2.1. Plant, Soil Samples, and Fungal Materials

*Lotus japonicus* Miyakojima MG20 and the *str* mutant [33] were used as plant materials. Twenty-four soils were collected from the field of Rakuno Gakuen University, Hokkaido, Japan. The original soil type of this area is Brown Forest soils [42]. Seventeen soils were obtained from non-cultivated sites that have not been used for cultivation at least for 5 years. Seven soils were obtained from sites that have been used for cultivation at least for 5 years. An AMF inoculum (*Rhizophagus* sp. strain R-10) was purchased from Idemitsu Kosan Co., Ltd., Tokyo. As shown in Table S1, some of the soil has been supplemented with soil from other places; there are also differences in the presence or absence of vegetation that can be a host for AMF.

### 2.2. Plant Growth and AMF Inoculation

Seeds of *L. japonicus* were scarified with concentrated sulfuric acid for 30 min and rinsed with deionized water five times, and immersed in deionized water overnight at 4 °C. The germinated seeds were grown in 100 mL polypropylene pots. The soil consists of 20 mL (bottom layer) of Akadama soil (tuff loam) (Setogahara Kaen, Gunma, Japan) and 80 mL of sample soils as upper layer. Plants were inoculated with AMF inoculum by mixing 1 g inoculum throughout the upper soil mixture before planting. Its inoculation potential was such that the formation of four infection units (an internal mycelium arising from entry points [43] were found in the roots of one plant 15 days after inoculation. The method of the detection of infection units was described previously [44,45]. For the control treatments, the same amount of unprocessed carrier (AMF-free) provided from the manufacturer was used. Pots were placed in a flat-bottomed tray in a growth chamber under 16 h light/8 h dark photoperiods (26 °C light /23 °C dark). Water was supplied from the bottom by maintaining a water level up to 5 mm in depth. Three WT and three mutants were planted in parallel in the same pot. Roots were severed from plants, carefully washed with water to remove the soil, grouped together, immediately immersed in 50% ethanol, and placed at 4 °C until use.

### 2.3. Fungal Cell Wall Staining

Roots were cleared with 10% [weight/volume (*w*/*v*)] potassium hydroxide (KOH) by boiling for 10 min, and then rinsed 5 times with water and once with phosphate buffered saline (PBS; pH 7.5). Roots were then immersed in 5 mL PBS containing 0.5% (*w*/*v*) skim milk (Wako, Osaka, Japan) and 0.4 μg·mL^−1^ wheat germ agglutinin (WGA)-conjugated horseradish peroxidase (HRP) (Vector, Burlingame, CA, USA). Roots were kept in this solution for more than 16 h at room temperature before being rinsed twice with PBS and then immersed in 5 mL PBS containing 0.2 mg·mL^−1^ DAB (3,3′-diaminobenzidine) tetrahydrochloride (Nakarai Tesque, Kyoto, Japan) and 0.1 μL·mL^−1^ 30% H_2_O_2_. The roots were kept in the DAB solution for 1 h at room temperature and then soaked in Tris–ethylene diamine tetra acetic acid (EDTA) (TE) buffer (10 mM Tris-HCl, 1 mM EDTA; pH 8.0) to stop the HRP reaction. Images were obtained using a stereomicroscope (SZX16 or SZ61, Olympus, Tokyo, Japan) equipped with a charge-coupled device camera.

### 2.4. Percent Root Length Colonization

The percentage of AMF-colonized root lengths was determined by a modified line intersect method [46] using a stereomicroscope. Briefly, roots were placed in 90-mm petri dishes that had parallel gridlines at 5-mm intervals, and root-gridline intersections were observed at 20× using a stereomicroscope (SZX16). A total of 200 intersections were analyzed in each batch of treatments. At each intersection, the presence of AMF intraradical hyphae were scored [44].

## 3. Results and Discussion

### 3.1. The Performance of Indigenous AMF Varies Among Soils

**Figure 1:**
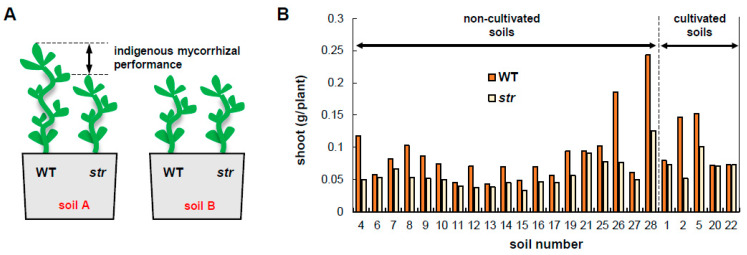
Bio-assay for evaluating the performance of indigenous arbuscular mycorrhizal fungi. (**A**) The schematic representation of the bio-assay. Three wild-type (WT) plants and three *stunted arbuscule* mutant (*str*) plants are grown in the same pot. The difference in shoot biomass between WT and *str* are the reflection of the performance of indigenous arbuscular mycorrhizal fungi (AMF) in the soils. (**B**) Shoot weights of WT and *str* plants grown for 20 days. Seventeen soils were obtained from non-cultivated sites that have not been used for cultivation at least for 5 years. Seven soils are obtained from sites that have been used for cultivation at least for 5 years.

To ensure that the soil conditions for growing WT and *str* plants were identical, three WT and three *str* seedlings were grown in the same pot (Figure 1A). The establishment of AMF colonization in the symbiotic manner (i.e., with formation of arbuscules) was observed in WT roots about 10 days after the start of cultivation. In all soils, the shoot biomass of WT plants was greater than that of the *str* mutant. This implies that mycorrhization with indigenous AMF did not have a negative effect on plant growth in these soils. It should be noted that the seeds of *str* are slightly larger than those of the WT plant, and there is no evidence that *str* are inferior to the WT regarding germination and initial growth at least two weeks after germination. The ratio of the shoot biomass of WT to that of *str* (indigenous mycorrhizal performance) ranged widely from 1 to 2.8; there was no trend in the ratio between cultivated soils and non-cultivated soils (Figure 1B).

### 3.2. Indigenous Mycorrhizal Performance is not Related to the Early Colonization Potential of Indigenous AMF and the Levels of P in the Soil

**Figure 2:**
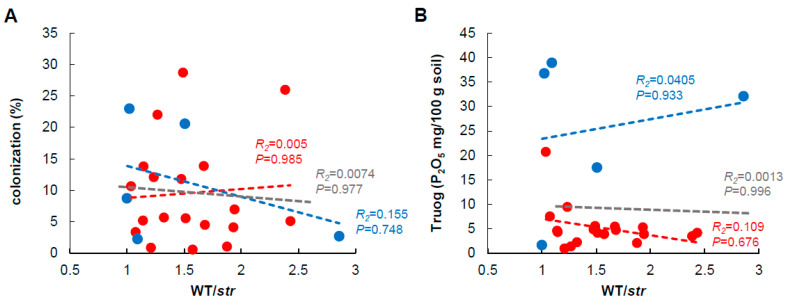
Indigenous mycorrhizal performance is not related to the early colonization potential of indigenous arbuscular mycorrhizal fungi and the soil phosphate levels. (**A**) The relationship between the performance of indigenous arbuscular mycorrhizal fungi (AMF) and the colonization potential of indigenous AMF in the soil. To evaluate the performance of indigenous AMF in the soils, we examined the growth ratios of wild-type and *str* mycorrhizal mutant using 24 soils. (**B**) The relationship between the performance of indigenous AMF and the level of phosphate (P) in the soil (Truog-P). Red- and blue-dotted lines are the regression lines calculated from the values of non-cultivated soils and cultivated soils, respectively. Gray-dotted lines are the regression lines calculated from all data.

To determine whether the difference in indigenous mycorrhizal performance was caused by the extent of AMF colonization, we examined the percent root length colonization by indigenous AMF at 20 days. The colonization levels of indigenous AMF in WT roots ranged from very low (<3%) to about over 20% in the soils of both cultivated fields and non-cultivated fields (Figure S1A). There was no statistically significant relationship between the indigenous mycorrhizal performance and the percent root length colonization in the soil of either cultivated and non-cultivated fields (Figure 2A). Notably, despite a very low indigenous mycorrhizal performance (1 < WT/*str* < 1.5), several soils exhibited high colonization levels. Conversely, some soils showed low colonization levels (<5%), despite a high indigenous mycorrhizal performance (Figure 2A). These results strongly support the previous findings that the effect of AMF on plant growth is independent of the extent of colonization [47,48,49].

The level of P in the soil varied greatly among soils (Figure S1B). In general, P levels were low in the non-cultivated soils and relatively high in the cultivated soils. This was clearly attributable to the presence or absence of previous fertilization. There was no relationship between soil P levels and indigenous mycorrhizal performance when the cultivated and non-cultivated soil samples were combined, and the overall P levels differed greatly (Figure 2B). Notably, some soils exhibited a low indigenous mycorrhizal performance in the presence of high colonization; a high indigenous mycorrhizal performance despite a low colonization level; and a high indigenous mycorrhizal potential at high soil P levels (Figure 2B). These responses are, as mentioned above, expected to vary in a context-dependent manner. In a future study, WT/*str* plants will be cultivated under various conditions at a small scale using these characteristic soils and the variation of their indigenous mycorrhizal performance will be investigated at the cellular and molecular level. It is expected that we can gain a more detailed understanding of the biological and physical factors influencing these mycorrhizal performances.

### 3.3. Relationship between Indigenous Mycorrhizal Performance and the Effect of Exotic AMF Inoculation

**Figure 3:**
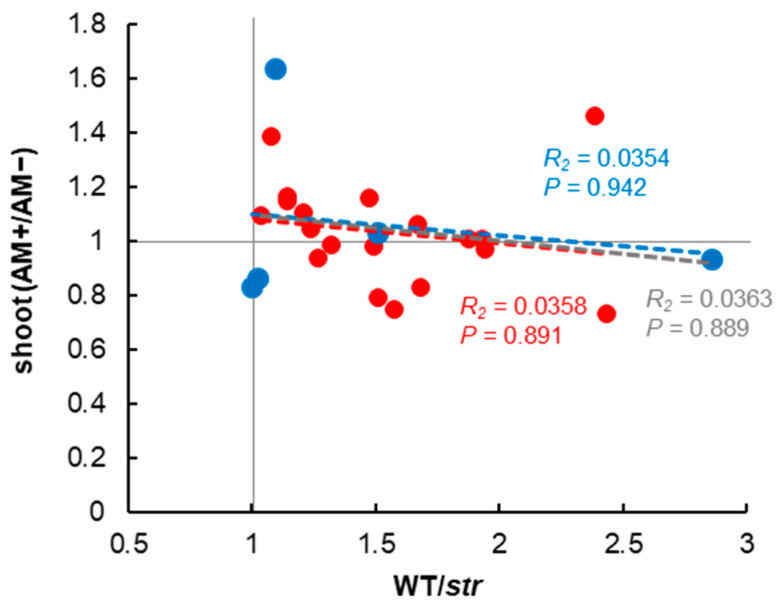
The relationship between the performance of indigenous arbuscular mycorrhizal fungi and the effects of the inoculation of exotic arbuscular mycorrhizal fungi in the soils. To evaluate the performance of indigenous AMF in the soils, we examined the growth ratios of wild-type and *str* mycorrhizal mutant using 24 soils. The effect of the inoculation of exotic AMF (Rhizophagus irregularis R-10) is shown by shoot weight ratio (inoculated/uninoculated). Red- and blue-dotted line is the regression line calculated from the values of non-cultivated soils and cultivated soils, respectively. Gray-dotted line is the regression line calculated from all data.

In soils with extremely low indigenous AMF performance, the outcome of the inoculation of exotic AMF may be more apparent [32]. Thus, we investigated the relationship between indigenous mycorrhizal performance and the growth-promoting effect of the inoculation of exotic AMF. Contrary to expectations, there was no statistically significant relationship between the indigenous mycorrhizal performance and the promotion of plant growth by inoculation (Figure 3). The dominance of the AMF inoculum (*Rhizophagus* sp.) used in this study in the roots was a necessary condition for its effectiveness in a field study [50]. Inoculation increased the colonization (from 0.8% to 1320%) in 15 out of 24 soils and decreased the colonization (from 14% to 100%) in nine out of 24 soils, suggesting that the inoculation had an effect on the colonization status (e.g., fungal composition or colonization dynamics). Regardless of the exact extent of inoculum AMF in the roots, the fact that the inoculation was ineffective implies that the soils with a low indigenous mycorrhizal performance may have specific biotic or abiotic characteristics that do not allow the expression of plant-growth promotion. Notably, however, it is possible that AMF play diverse roles beyond promoting plant growth [51,52]. In the present study, we used the *str* mutant, which exhibits a multifaceted loss of function; however, in the future, we may be able to evaluate the specific AMF function as indigenous mycorrhizal performance or inoculum by using individual functional mutant plants, such as phosphate transporters [53] and ammonium transporters [54].

Importantly, despite the fact that the growth ratio of WT to *str* did not indicate a negative effect of indigenous fungi on plant growth (Figure 1A), inoculation with exotic AMF altered the shoot biomass in both positive and negative ways. This suggests that, while mycorrhization does not negatively affect plant growth within the biotic and abiotic components from the same soil, the inoculation of exotic AMF into the soil ecosystem has a significant impact on the growth of plants. The introduction into the soil of exotic AMF with different life-story strategies compared with indigenous AMF may damage the indigenous fungi [8].

In this study, we assessed the effectiveness of the ratio of the growth of *L. japonicus* WT to that of the *str* mutant to evaluate the indigenous mycorrhizal performance in 24 soils. Each soil had its own unique indigenous mycorrhizal performance that was not directly related to the extent of colonization of indigenous AMF or the P level in the soil. The low indigenous mycorrhizal performance could not be compensated by the inoculation of exotic AMF. Indigenous mycorrhizal performance did not become negative. However, the inoculation of exotic AMF into the same soil could have both positive and negative effects. In agriculture, horticulture, and home gardening, adding soil from different places and transplanting soil-soaked plants are performed often. The AMF contained therein may also affect the subsequent growth of the plant for better or worse, at least in the short-term. The results of this study suggest for the first time that indigenous mycorrhizal fungi are harmless to plants in their native soil ecosystems. The investigation of the mechanisms underlying the impact of exotic AMF inoculum on plant growth or nutrition, for better or worse, is a future challenge.

## Supplementary Materials

The following are available online at https://www.mdpi.com/2223-7747/9/5/658/s1, Table S1: The biological and chemical properties of 24 soils used in this study. Figure S1: The colonization potential of indigenous arbuscular mycorrhizal fungi and phosphorus content in the soils used in this study. (A) Colonization potential of indigenous arbuscular mycorrhizal fungi (AMF) of 24 soils. *Lotus japonicus* wild-type seedlings were grown for 20 days in pot culture. (B) Phosphate levels of 24 soils determined by Truog method. Red-bars and blue-bars indicate the soils collected from non-cultivated fields and cultivated fields, respectively.
