# Supplementary material for: Investigation of Indigenous Arbuscular Mycorrhizal Performance Using a Lotus japonicus Mycorrhizal Mutant
Source: Plants (Basel). 2020 May 22;9(5):658. doi: 10.3390/plants9050658 (PMC7284865; doi:10.3390/plants9050658)
Supplement: Supplementary file 1 [file plants-09-00658-s001.zip › 0506_Teranishi&Kobae_supple.pdf]

Supplementary Material

soil	type	vegetation	introduction of different soil	Truog (P_2O_5 mg/soil 100g)	colonization (%)
1	cultivated	oat	yes	38.9	2.2
2	cultivated	corn	yes	32.1	2.7
4	non-cultivated	yes	yes	3.5	26.0
5	cultivated	n.d.	yes	17.5	20.6
6	non-cultivated	no	no	7.5	3.4
7	non-cultivated	no	no	9.4	12.1
8	non-cultivated	no	no	5.3	4.1
9	non-cultivated	no	no	4.7	4.5
10	non-cultivated	yes	no	5.6	28.7
11	non-cultivated	yes	yes	4.3	13.8
12	non-cultivated	no	yes	2.1	1.1
13	non-cultivated	yes	no	4.6	5.2
14	non-cultivated	yes	no	3.9	0.6
15	non-cultivated	yes	no	4.9	11.8
16	non-cultivated	yes	yes	4.2	5.6
17	non-cultivated	yes	yes	1.4	22.0
19	non-cultivated	no	yes	5.4	13.8
20	cultivated	n.d.	no	36.7	23.0
21	non-cultivated	no	yes	20.7	10.6
22	cultivated	flower	yes	1.7	8.7
25	non-cultivated	no	no	2.2	5.7
26	non-cultivated	yes	no	4.2	5.1
27	non-cultivated	no	no	1.0	0.9
28	non-cultivated	yes	no	3.9	7.0

Table S1
The management history of 24 soils used in this study

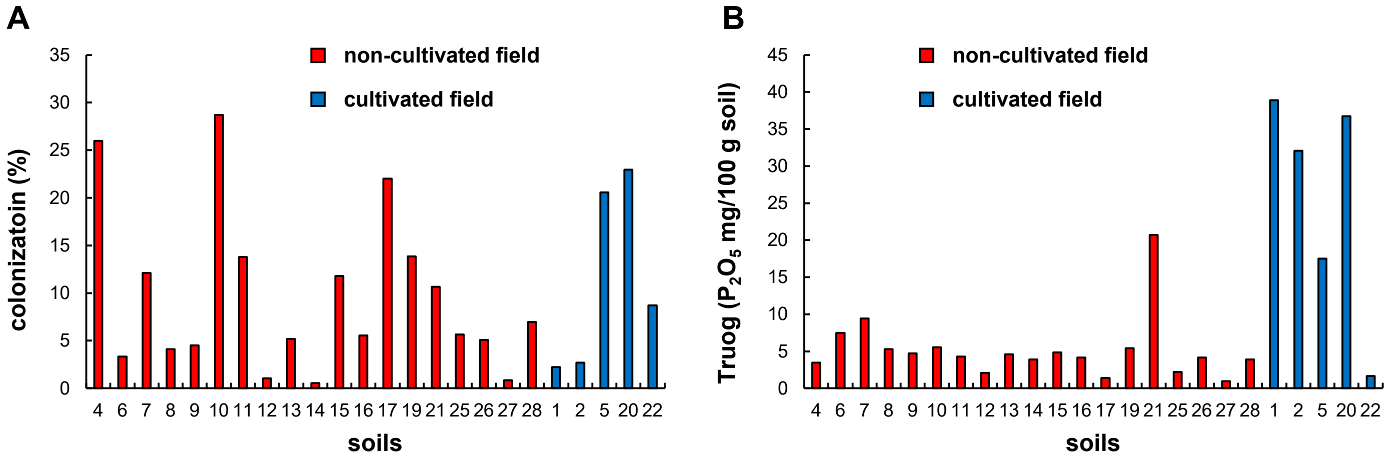

Figure S1
The colonization potential of indigenous arbuscular mycorrhizal fungi and phosphorus content in the soils used in this study. (A) Colonization potential of indigenous arbuscular mycorrhizal fungi (AMF) of 24 soils. *Lotus japonicus* wild-type seedlings were grown for 20 days in pot culture. (B) Phosphate levels of 24 soils determined by Truog method. Red-bars and blue-bars indicate the soils collected from non-cultivated fields and cultivated fields, respectively.
